# Host, pathogenic fungi and the microbiome: A genetic triangle in infection

**DOI:** 10.3389/fimmu.2022.1078014

**Published:** 2023-01-17

**Authors:** Sara Gago, Martina Mandarano, Claudia Floridi, Teresa Zelante

**Affiliations:** ^1^ Manchester Fungal Infection Group, School of Biological Sciences, Faculty of Biology, Medicine and Health, The University of Manchester, Manchester, United Kingdom; ^2^ Department of Medicine and Surgery, University of Perugia, Perugia, Italy

**Keywords:** Fungal infections, *Aspergillus fumigatus*, *Candida albicans*, microbiome, immunodeficiency

## The first perspective: The host genetics

The outcome of fungal disease is determined by complex interactions between fungal pathogens, human hosts and their environment including the host microbiome (1–4). Morbidity and mortality in fungal disease remain very high despite recent advances in the diagnostic and treatment of these conditions (5–7). There are only three classes of antifungal drugs available to treat these disease and, antifungal resistance linked to the use of agricultural use of triazole fungicides is on the rise (8). The development of new antifungal drugs to treat human fungal disease is challenging as both, host and pathogen are eukaryotes and, there are different potential druggable targets exposed at different points of fungal morphogenesis.

**Figure 1:**
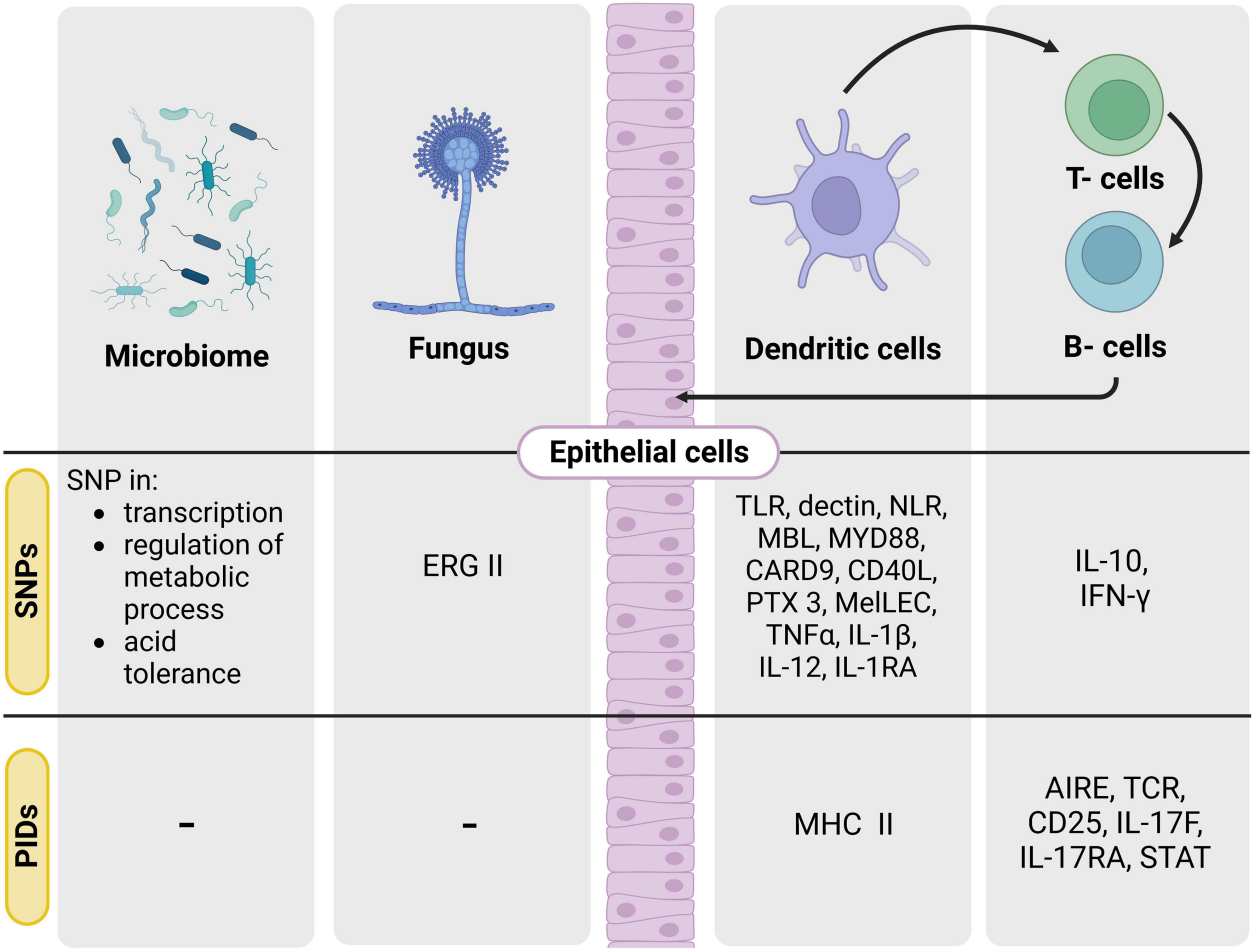
SNPs and PIDs known in the ‘genetic triangle’ leading to opportunistic human fungal infections. Scheme of the main target genes involved in SNPs and PIDs leading to the main described human fungal diseases. Single nucleotide polymorphisms (SNPs), primary immunodeficiencies (PIDs).

So far, the identification of high-risk patients for fungal disease has relied on the use of clinical scores that combine the use of clinical and host factors to predict the risk of subsequent disease (9–11). However, the prevalence of opportunistic fungal diseases within at-risk population, ranges from 0.1 – 20% (12). In the last decades, individual genetic variation has been recognised as a major contribution of functional immune responses against fungal pathogens. Several monogenic defects and polymorphisms in genes regulating antifungal immunity or pathogen sensing have been associated with susceptibility to aspergillosis, cryptococcosis and candidiasis (13) (**Figure 1**).

Sensing of human fungal pathogens by the host immune system requires the interplay between pathogen-associated molecular patterns (PAMPs), mostly located in the cell wall of fungal pathogens, and pattern recognition receptors (PRRs) (14–17). The interaction between PRRs and PAMPs, leads to the regulation of uptake of fungal pathogens by immune cells. In addition to membrane receptors, soluble PRRs such as pentraxins or mannose binding lectins (MBLs) are also critical for pathogen sensing and efficient phagocytosis (18, 19).

To date, polymorphisms in PTX3 have been reported in different clinical settings as a risk factor for invasive pulmonary aspergillosis in haematopoietic stem cell transplant recipients (20), solid organ transplants (21) and chronic obstructive pulmonary disease (22). Using *ex vitro* an *in vivo* models of disease it has been reported that PTX3 deficiency increases susceptibility to *A. fumigatus* infection due to impaired neutrophil function (20, 23).

Polymorphisms in PRRs and other immune pathways have been reported in different patient cohorts (24–28). Nevertheless, none of these polymorphisms (except in the case of primary immunodeficiencies) allow to predict risk of fungal disease with high specificity suggesting the genetic basis of these diseases may be polygenic.

Despite genetic replication studies are in general scarce, the link between genetic polymorphisms in PTX3 (rs1840680) and rs7309123 (CLEC7a) and aspergillosis risk have been successfully replicated (29). *White et al.* (30) recently explored whether screening for genetic variants in genes previously linked with susceptibility to invasive aspergillosis alongside clinical factors and mycological evidence could be used to improve aspergillosis risk stratification in patients undergoing allogeneic stem cell transplantation. In their model, they reported that mutations in Dectin-1, DC-SIGN, allogeneic stem cell transplantation, current respiratory viral infection and *Aspergillus*-specific positive PCR were all high-risk factors for the development of invasive disease.

An increasing number of case studies and family studies have reported fungal disease in children with primary immunodeficiencies. For example, invasive aspergillosis has been linked with inborn errors in patients with chronic granulomatous disease, severe congenital neutropenia or leukocyte adhesion deficiency type I (31, 32). Moreover, other less common congenital immunodeficiencies (e.g., CARD9 immunity, IL-12/interferon (IFN)-γ axis or IL-17 immunity) have been described to increase susceptibility risk to invasive candidiasis, dermatophytosis, chronic mucocutaneous candidiasis or endemic mycoses (**Figure 1**) (33–36).

Genome-wide association studies (GWAS) have allowed us to identify a number of novel genetic loci affecting susceptibility to fungal infections (37, 38). A GWAS study of patients with common infections revealed a significant association between *DSG1* variants and susceptibility to vulvovaginal candidiasis. *DSG1* encodes for a desmoglein, a critical protein involved in maintaining the integrity of the epithelial compartment (37, 39).

GWAS in patients with candidemia revealed a strong association between the genetic variant rs8028958 in the *PLA2GB4* gene and susceptibility to disease (40). *PLA2GB4* encodes a cytosolic phospholipase A_2_ involved in lipid metabolism, affecting cytokine production in the presence of *Candida* in the bloodstream (40).

Combining the use of GWAS, bulk RNA-seq and scRNA-seq from human PBMCs upon *Candida* stimulation, a recent study suggested a critical role of *LY86* in susceptibility to candidemia. *LY86* encodes for *Lymphocyte Antigen 86*, mainly expressed in monocytes. *LY86* silencing impairs monocyte migration, increasing susceptibility to candidemia (38).

The role of genetic variation in genes encoding for host cytokine responses has been extensively studied including data in the Human Functional Genomics Project (41). In this study, 17 new genome-wide significant loci that influence cytokine production were identified (41). *In vitro* studies of human PBMCs challenged with fungi demonstrated a high inter-individual variation in cytokine release (IL-6, TNF-α, IL-1β) (39). Thus, suggesting many genome-wide quantitative trait locus (QTLs) might contribute to susceptibility to infectious. Interestingly, this study shows that the QTLs are not affecting adaptive cytokines as IL-17 (41).

How far are we from implementing host genetic screening in the diagnostic pipelines for fungal disease? Studies aiming to characterise the genetic basis of fungal disease have been based on association studies with either disease and common polymorphisms in genes known to be important for efficient antifungal responses such as those involved in antigen presentation, pathogen sensing, or regulation of immune pathways. Even though these associations are not surprisingly significant, they are present in the general population. In addition, rigorous clinical definitions for some diseases such as allergic and chronic forms of aspergillosis or more recently viral-associated fungal disease have not been available until recently thus, hampering the usefulness of genetic risk to predict susceptibility to fungal disease. To overcome this issue, whole genome exome or genome sequencing studies might be useful (42–44). However, a joint effort from the scientific community should be made to optimise and simplify bioinformatic pipelines. Finally, implementation of host genetic screening in the diagnostic pipelines for fungal disease would require validation in large and well-characterised cohort of patients with different genetic backgrounds and the development of point of care testing approaches that would allow the transference of these technologies to those regions where the prevalence of fungal disease is particularly high.

## The second perspective: The pathogen genetics

Most of what we know about the pathogenicity mechanisms used by fungal species to cause disease has arisen from *in vivo* or *in vitro* infection models in which a particular fungal species, clinical strain or deletion mutant is assessed for virulence. However, results are very much dependent on the animal strain used, the model of disease (e.g., immunosuppression *vs* no immunosuppression), the dose or route of infection, or the cell population assessed thus, results are not always translated into human disease. In addition, to understand the opportunistic nature of most fungal human pathogens, it is important to consider that genetic drivers of virulence have probably been developed so fungal pathogens can survive in their natural environments (45). In fact, in a recent publication using population genomics, it was observed that human infections caused by drug resistant *A. fumigatus* have their origin in the environment (46).

With the increasing number of sequenced fungal genomes it has been observed that pathogenicity emerged in different lineages in the fungal kingdom (43). However, there is a huge variation in fungal drivers of human disease among pathogens but also strains from the same pathogen. For example, virulence of *A. fumigatus* strains is significantly different depending on the infection model of disease used (47). Nevertheless, it seems that there is a link between the capacity of a fungal pathogen to adapt to extreme environments and their capacity to cause disease.

The human mould pathogen *A. fumigatus* can cause invasive, chronic or allergic diseases in immunosuppressed patients or those with a chronic respiratory condition (48). In fact, Snelders et al. (49),using whole genome sequencing of fungal isolates from patients with cystic fibrosis and chronic pulmonary aspergillosis demonstrated than parasexual recombination is critical for *A. fumigatus* adaptation and might also be a driver for the development of azole resistance beyond the occurrence of point mutations in the *CYP51* (Erg11) (**Figure 1**) (50). Similarly, Ballard et al. (51) reported that long-term *Aspergillus* infection in patients with chronic granulomatous disease is driven by host microevolution (51). Moreover, recent analyses of fungal pangenomes has shown that *A. fumigatus* environmental isolates do not differ in their gene content (52). However, it has been shown an increased number of accessory genes in clinical isolates compared to environmental that might help to better understand human disease (53).

Fungal species of the same genera can also cause disease to different populations as for *Cryptococcus neoformans* and *Cryptococcus gattii*. However, within each of these species there is a significant genomic and phenotypic heterogeneity (53, ) that can be linked with disease outcomes. Similarly, there is a significant genomic and phenotypic variability within *C. albicans* and some loss of function mutations might help to better understand genetic drivers of disease (54).

Recently, we have discovered a new model where the metabolic route of tryptophan degradation, as well as the total amount available of tryptophan, differently affect fungal virulence. Fungi express the tryptophan degrading enzyme Indoleamine 2,3-dioxygenases that degrade l-tryptophan to kynurenines. *Aspergillus fumigatus* possesses three *ido* genes that are expressed under hypoxia or tryptophan abundance. Loss of *ido* genes increases fungal pathogenicity due to the activation of the tryptophan-degrading enzyme AroH (55).

## The third wheel: The host microbiome genetics

Until recently, the contribution of the environment to the development of fungal diseases has been mainly linked to ecological factors such geographic distribution, climate or the existence of a possible zoonotic reservoir (45, 56). However, there is an increasing number of studies suggesting that the host microbiome, is crucial in driving resistance against fungal disease (57). In particular, host xenobiotic receptors (XRs) activated by metabolism may affect susceptibility to fungal infection (58, 59). Indeed, several factors such as tissue microenvironment, diet, nutrient availability or antibiotic exposure are known to affect the microbiome evolution and microbial SNPs (60, 61). Probiotics may also acquire SNPs when several stressors act in particular microbiome niches (**Figure 1**) (62).

Fungal*-*bacteria interactions in clinically relevant contexts such as oral, gut and respiratory dysbiosis have been increasingly studied and, both synergistic and antagonistic interactions have been reported. Several studies have shown that *Candida albicans* germination and virulence can be directly or indirectly regulated by bacteria such as *Lactobacillus* spp. For example, lactobacilli release quorum sensing molecules or antifungal molecules (e.g., hydrogen peroxide or organic acids) to prevent fungal growth (63). Similarly, we recently found that *Lactobacillus reuteri* reduces *C. albicans* gut colonization *via* metabolic activation of specific bacterial gene cluster and the release of indole-derivatives (59). A similar phenotype has been shown in vulvovaginal candidiasis (64). In oral mucositis, *Candida* spp adheres to *Streptococcus* biofilms by increasing the expression of *Als1* or *Als5* genes (65). In addition, *C. albicans* and *S. mutants* interact in biofilm formation in which *C. albicans*-induced expression of *S. mutans glucosyltransferase B*, facilitating pathogen-pathogen binding (66, 67). *L*. *crispatus* SNUV220 and *L*. *fermentum* SNUV175 supernatant downregulates the expression of the hypha-related genes *ALS3*, *ECE1*, *SAP5* and *HWP1* in *C. albicans (* 68 *).* In an independent study, it was shown that *L. plantarum* SD5870, *L. helveticus* CBS N116411 and *S. salivarius* DSM 14685 also inhibit *Candida* yeast-hypha transition (69).

**Figure 2:**
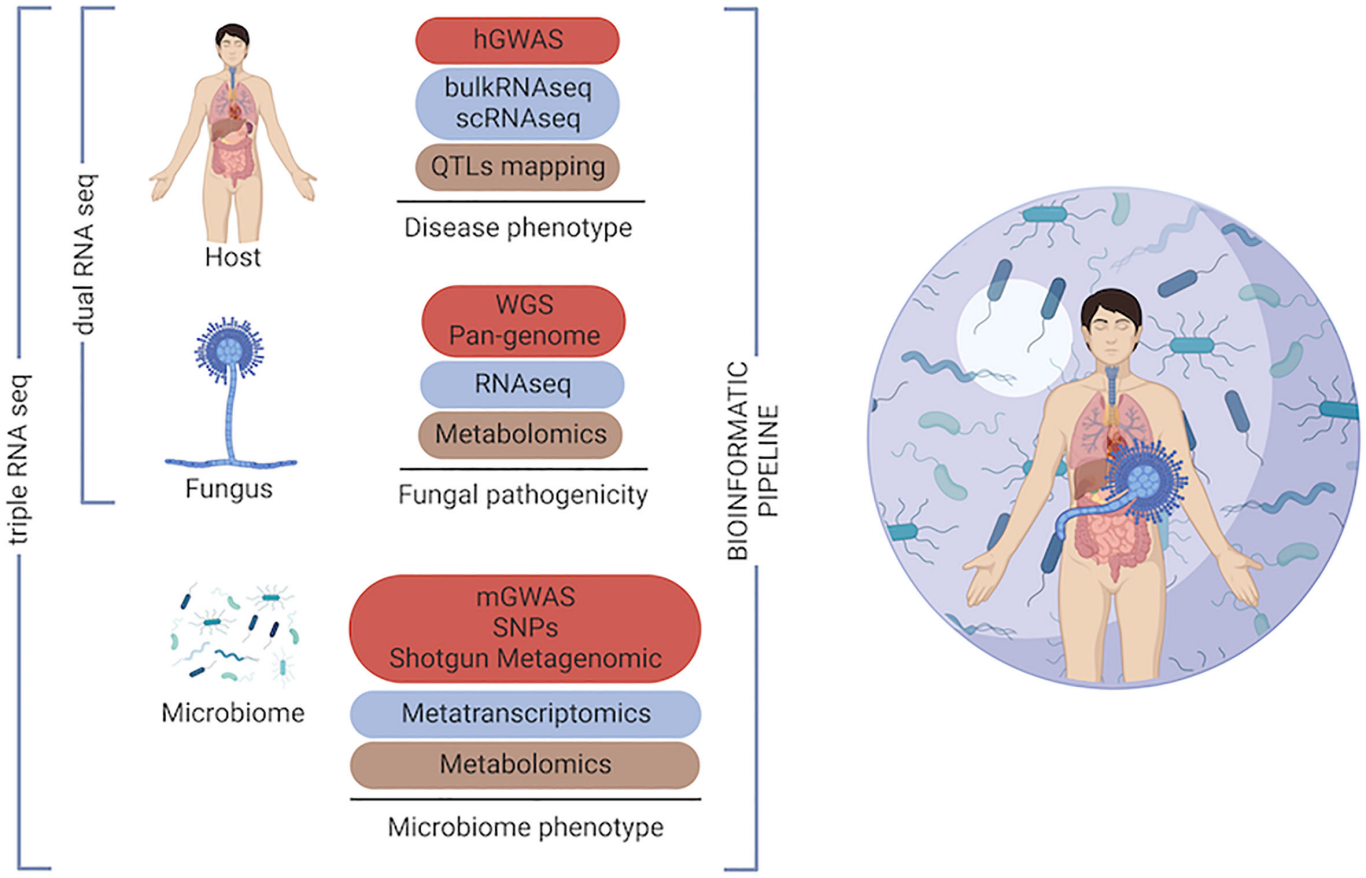
Omics toolkit for investigating the ‘genetic triangle’ in the human host during fungal infection. Multi-omics approaches, which enable intermediate phenotypes into the host, the fungus and the host microbiome to be measured by different -omics technologies. Genome-wide association studies (GWAS), quantitative trait locus (QTLs), single nucleotide polymorphism (SNP).

The combinatorial impact of host genetic variation and pathogen genetics in the outcome of fungal disease has been overlooked. Only recently, these two approaches have been integrated by using the dual RNA sequencing in infectious diseases (70). This approach, that has previously been used for plant-host interaction studies, consists in performing parallel transcriptomic analysis of pathogens and their eukaryotic host cells (71). Thus, multi-organism RNA-seq, may be applied to the human population bearing opportunistic fungal infections, eventually co-infected with other pathogens. Moreover, human genetic variants may be analysed alongside fungal genetic variability by using integrated GWAS approaches as reported for meningitis (72) and, it could be potentially expanded to define microbiome genetic variants. This systems biology approach will enable us to define the role of genetic variation in the host, microbiome and the pathogen with a view to improving our understanding of the complexity of the human ecosystem during infection (**Figure 2**).

## Author contributions

MM, CF critically read, analyzed, and discussed the literature and conceived the outline of the manuscript. SG and TZ wrote the manuscript. All authors contributed to the article and approved the submitted version.
